# Challenges in lung cancer screening access among socioeconomically distressed communities

**DOI:** 10.1016/j.xjon.2025.09.011

**Published:** 2025-09-18

**Authors:** F.N.U. Nandni, F.N.U. Venjhraj, Mukesh Kumar, Sangeeta Davi

**Affiliations:** a Jinnah Sindh Medical University, Karachi, Sindh, Pakistan; b Shaheed Mohtarma Benazir Bhutto Medical College Lyari, Karachi, Sindh, Pakistan; c Peoples University of Medical and Health Sciences For Women (PUMHSW), Nawabshah, Sindh, Pakistan

To the Editor:

**Figure:**
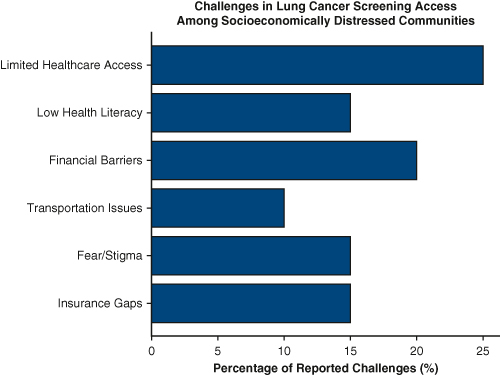

We recently reviewed the article by Woods and colleagues,^1^ in which they explore disparities in lung cancer screening among individuals residing in socioeconomically disadvantaged areas. This timely investigation offers meaningful insights into the relationship between socioeconomic status, as measured by the Distressed Communities Index (DCI), race, and access to screening within a high-volume clinical setting. The authors are to be commended for their thorough analysis and valuable findings, notably the identification of Hispanic and Asian populations as underrepresented in screening programs, and the important observation that screening outcomes were consistent across both racial and socioeconomic groups.

However, we would like to share some insights and situate these findings within the broader context of lung cancer screening disparities. First, this study highlights lower lung cancer screening rates among Hispanic and Asian minorities, even among patients from highly distressed communities. This reflects earlier evidence that current screening guidelines and health care systems often fail to adequately reach high-risk racial and ethnic groups. Black and Hispanic populations, in particular, are disproportionately underrepresented due to strict pack-year eligibility criteria and additional barriers such as language, cultural influences, and structural racism.^2^ To close these gaps, culturally informed outreach strategies and greater provider awareness remain essential.

Second, the authors’ use of the DCI as a community-level socioeconomic measure provides an innovative approach to assessing disadvantage beyond single factors such as income or education. Previous studies demonstrate that low socioeconomic status is linked to greater lung cancer incidence and mortality, largely as the result of greater smoking prevalence, greater comorbidities, delayed diagnosis, and inequities in treatment access.^3^ Notably, the finding that malignancy detection and clinical outcomes were similar across DCI groups supports emerging evidence that disparities are rooted primarily in access to screening rather than in treatment once screening is achieved.^4^

Third, the single-center, retrospective nature of this study may restrict the generalizability of its findings, because screening access can vary widely across geographic regions and institutions. Nationally, rural residents and Medicaid-insured populations face notable obstacles, including limited geographic availability, insurance-related challenges, and social determinants that shape both screening participation and outcomes.^3^ Broader studies across diverse settings, while also accounting for patient-level barriers beyond community indices, are needed to more fully capture the complexity of screening disparities.

Lastly, the study draws attention to persistent limitations in guideline eligibility, particularly the dependence on pack-year smoking history, which disproportionately excludes minority populations who often develop lung cancer despite lighter smoking exposure.^5^ Emerging evidence indicates that risk-based models for lung cancer screening offer greater equity and efficiency than traditional criteria,^E1^ and their adoption could further expand access for underserved groups.

In conclusion, this study provides important insights into disparities in lung cancer screening, underscoring challenges in reaching minority populations, the value of socioeconomic measures, and the impact of geographic and institutional differences. Their findings also highlight the potential of risk-based models to enhance equity. Ongoing efforts to refine guidelines, expand culturally informed outreach, and broaden research will be vital to ensuring screening benefits are equitably shared. Thank you for considering this perspective.

## Conflict of Interest Statement

The authors reported no conflicts of interest.

The *Journal* policy requires editors and reviewers to disclose conflicts of interest and to decline handling or reviewing manuscripts for which they may have a conflict of interest. The editors and reviewers of this article have no conflicts of interest.
